# Influence of *Morus alba* Leaves Extract on Human Erythrocytes

**DOI:** 10.3390/biology14081005

**Published:** 2025-08-05

**Authors:** Stefano Putaggio, Annamaria Russo, Giuseppe Tancredi Patanè, Antonella Calderaro, Santa Cirmi, Ivana Verboso, Giuseppina Laganà, Silvana Ficarra, Davide Barreca, Françisco Raymo, Ester Tellone

**Affiliations:** 1 Department of Chemical, Biological, Pharmaceutical and Environmental Sciences, University of Messina, Viale Ferdinando Stagno d’Alcontres 31, 98166 Messina, Italy; stefano.putaggio@studenti.unime.it (S.P.); giuseppe.patane@studenti.unime.it (G.T.P.); anto.calderaro@gmail.com (A.C.); santa.cirmi@unime.it (S.C.); giuseppina.lagana@unime.it (G.L.); silvana.ficarra@unime.it (S.F.); davide.barreca@unime.it (D.B.); etellone@unime.it (E.T.); 2 Virology and Microbiology AOOR Papardo-Piemonte, 98166 Messina, Italy; ivimel@hotmail.it; 3 Laboratory for Molecular Photonics, Department of Chemistry, University of Miami, Coral Gables, FL 33146-0431, USA; fraymo@miami.edu

**Keywords:** erythrocytes, nutrients, cell viability, cell metabolism, oxidative stress

## Abstract

*Morus alba* L. (MA) is a member of the Moraceae family, known as “white mulberry”. Due to the high levels of bioactive compounds, mulberry plants can be considered a good source of nutrients and antioxidant compounds. Our study aims to analyze the effect of MA extract leaves on erythrocytes, focusing on its action on metabolism and membrane integrity. The choice of erythrocytes as a study model is based on their metabolic simplicity and their easy availability. Cell viability, following exposure of the cells to the extract, was evaluated by hemolysis, methemoglobin, caspase 3 activity and flow cytofluorimetric analysis; in addition, the effect of the pretreatment with the MA was detected after incubation of erythrocytes with different stressors. The impact on cell metabolism was evaluated by measuring anion flux kinetics, ATP levels and phosphatase activity. The results obtained show a peculiar (double) effect of the extract, which, on the one hand, probably by exploiting its component with antioxidant properties, protects the cell membrane by accumulating on the bilayer. On the other hand, the alteration of anion exchange could lead to the triggering of apoptosis and consequent cell death. The hypotheses, although excluded by our data, all point toward a beneficial and protective action of the extract on the health and vitality of RBCs.

## Simple Summary

In recent years, research into natural bioactive compounds has been recognized as a milestone in biomedical research, driven by an increasing focus on their potential therapeutic benefits and contributions to human health. In this view, *Morus alba* L. (MA), being a good source of antioxidant compounds, could be a promising supplementation of erythrocyte stored bags with natural compounds. The study on the effect of *Morus alba* extract on erythrocytes shows a peculiar effect of the extract, which, on the one hand, probably by exploiting its component with antioxidant properties, protects the cell membrane by accumulating on the bilayer. On the other hand, the alteration of anion exchange could lead to the triggering of apoptosis and consequent cell death. The hypotheses, although excluded by our data, all point toward a beneficial and protective action of the extract on the health and vitality of RBCs. These controversial effects of *Morus alba* extract pave the way for new, more in-depth studies on the potential use of this plant’s intriguing properties, which, if well understood and targeted, could increase the basal antioxidant protection load and shelf life of erythrocytes.

## 1. Introduction

*Morus alba* (MA) is a plant that belongs to the Moraceae family, known as the “white mulberry”; it is native to the central and northern regions of China and was introduced in Italy for silkworm breeding [1]. Today’s MA plant is present in almost all regions of Italy, where it is cultivated as a fruit plant, even if its fruits are not easily preserved. The scientific name of *Morus alba* derives from the Greek “Meros” (part) in reference to small fleshy fruits and “Albus” (white) in reference to the color of the fruits. It is precisely the sweetness of its fruits that has rapidly increased its cultivation and consumption, while the leaves of the plant are frequently used in the production of traditional Chinese medicines. Mulberries, due to their high levels of bioactive molecules, can be considered a good source of nutrients and antioxidant compounds (such as polysaccharides, flavonoids, polyphenols, vitamins, amino acids, anthocyanins). The leaves have been analyzed for their composition in flavonoids and bioactive compounds, as reported in the literature [2,3]. Dugo et al. reported the isolation and identification of 22 compounds in the hydroalcoholic extract of leaves and de Oliveira showed the presence of chlorogenic acid [4,5]. Moreover, MA plants are rich in 1-deoxynojirimycin (DNJ), a glucose analogue with a secondary amino group instead of an oxygen atom in the glucose pyranose ring; its presence is commonly used as a reference to assess the quality of the plant material and standardize the extracts, as reported by Marchetti et al. [6,7]. DNJ molecules likely play a key role in mulberry’s antidiabetic effect due to their strong inhibition of α-glucosidase in the small intestine; DNJ also improves insulin sensitivity, reduces inflammation through multiple mechanisms and shows antioxidant properties, reducing oxidative stress [8,9,10]. In vitro and animal model studies have shown that MA extracts can improve insulin sensitivity and positively influence lipid and carbohydrate metabolism. In addition, the extract has anti-inflammatory, anti-aging and antioxidant properties [11,12,13,14,15].

Red blood cells (RBCs), being the main constituents of blood, are naturally distributed throughout the body by the circulatory system, where access to various tissues makes them an important tool for assessing the health of the body. They are particularly exposed to environmental stimuli, and their continuous contact with exogenous molecules to which they react through metabolic adaptations can be a useful biochemical index of environmental impact [16,17]. Erythrocytes, in addition to their primary function of transporting oxygen from the lungs to tissues and carbon dioxide from tissues to the lungs, are involved in the buffering of excess hydrogen ions in the body. Also, they contribute to the maintenance of cardiovascular homeostasis thanks to their additional functions, such as the metabolism of nitric oxide (NO) and the control of blood rheology; moreover, RBCs perform an erythrocrine function, i.e., they have the ability to release signal molecules, including NO, metabolites and adenosine triphosphate (ATP) [18]. While considering the significant relevance of these cells, it is important to underline that RBCs are relatively simple from a metabolic point of view, and this is due to the absence of a nucleus that limits their metabolic development. In particular, the lack of mitochondria, by reducing the complexity of the cell, simplifies analysis and allows researchers to study specific metabolic pathways. RBCs are mainly based on glycolysis for energy production; this simplifies the study of the impact of molecules on their cellular metabolism. In addition, the red blood cell membrane is a well-defined structure, which gives the cell a particular flexibility by allowing it to navigate through narrow capillaries. These mechanical characteristics of the membrane and its relative ease of insulation also make RBCs a good model for the study of membrane structure, function and deformability. Finally, blood is easily obtainable, and RBCs are also easily accessible and isolated, making them a cost-effective choice for both research and diagnostic purposes. Therefore, the conduct of in vitro tests using human RBCs obtainable, building of simplified models for metabolic studies.

Considering the above-mentioned aspects, it may be interesting to analyze the cytoprotective MA effects on the metabolism of isolated RBCs. In fact, the natural origin of MA extract makes it particularly attractive for therapeutic applications; however, despite studies having offered valuable insights into its properties on some cellular models, there is a noticeable lack of experimental evidence of the impact of MA on erythrocytes [19]. Few data, as reported above in the literatre, suggest MA as an ingredient for the creation of functional foods with good application prospects, demonstrating its protection on the RBC membrane from oxidative damages [20,21]. Therefore, this study will contribute to a better understanding of the beneficial effects of MA leaf extract, evaluating its impact on human erythrocyte metabolism and membrane integrity.

## 2. Materials and Methods

### 2.1. Reagents and Compounds

All reagents used for the experiments were purchased from Sigma Aldrich (St. Louis, MO, USA). Human blood was obtained from 20 healthy, conscious volunteers (10 males and 10 females), between the ages of 27 and 30, with normal MDI range, no drug use in the last month, and who had followed a Mediterranean diet. Informed consent was obtained from all subjects involved in the study. Erythrocytes were stored for up to 24 h from the withdrawal in tubes containing ethylenediaminetetraacetic acid (EDTA) (as an anticoagulant) and subsequently used for experiments at 4 °C. The approval of the study was obtained by a Local Ethics Committee (prot. 71-23 of 5 April 2023) in accordance with the Declaration of Helsinki. The leaves belonging to *Morus alba* L. were purchased at the local market in June–August 2024 and identified by a systematic botany expert. Macroscopic analysis of the MA leaves used in the following study revealed that they have an ovate shape and a cordate base, that is, they have two rounded lobes that join. The adaxial surface of the leaf (upper face) is shiny, while the abaxial surface (lower face) has scattered hairs on the main veins. The margins of the leaf are instead serrated.

### 2.2. Preparation of Morus alba Extract

The leaves of MA were dried inside an oven, at a temperature of 50 °C, until a humidity value of 2% was obtained, and then, using a mortar, they were ground and passed through a sieve (80.0 mm mesh). The powder (1.0 g) was treated, for extraction, with a solution of ethanol and water (70:30%, *v*:*v*, respectively), under agitation at 25 °C, for 12 h in the ratio 1:100 (*w*:*v*). Afterwards, the solution was centrifuged at 10,000 rpm for 5 min, and the supernatant was filtered using a paper filter. The remaining powder was again extracted with the same amounts of solvent, as reported above, and the operation was repeated 3 times. Subsequently, using a rotavapor, the sample was concentrated to a final volume of about 5 mL, collected and dried into powder using lyophilization. The powder was diluted with distilled water, and the resulting sample was normalized to 2% of 1-deoxynojirimycin according to European Pharmacopeia, as determined by HPLC separation, obtaining a solution with a final concentration of 1.0 mg/mL.

### 2.3. Chemical Characterization of the MA Components by Quantification of Phenolic, Flavonoidic and Carbohydrate Concentration Present in the Extract

#### 2.3.1. Phenolic Concentration

The content of phenolic concentration was evaluated by using the Folin–Ciocalteu reagent method by spectrophotometry [22]. Briefly, the extract (100.0 μg/mL) was stirred with Folin reagent (10%) for 10 min, and then with Na_2_CO_3_. (20%) for 20 min. The concentration of phenolic compounds in the extract was evaluated at 755 nm, with a Beckman DU 640 spectrophotometer (Harbor Boulevard, Fullerton, CA, USA), and expressed as mg gallic acid equivalents.

#### 2.3.2. Flavonoid Concentration

Total flavonoid content in the extract was evaluated by the aluminum chloride colorimetric method [23]. The extract (100.0 μg/mL) was mixed with AlCl_3_ (10%), methanol, CH_3_CO_2_K (1 M) and H_2_O. After 30 min incubation, the absorbance value was evaluated at 415 nm using a Beckman DU 640 spectrophotometer (Harbor Boulevard, Fullerton, CA, USA). The total flavonoid content was expressed as mg quercetin equivalents.

#### 2.3.3. Carbohydrate Concentration

The determination of sugars was performed as reported by Miller [24]. DNSA reagent was prepared by mixing 500.0 mL NaOH (2%) with 10.0 g of dinitrosalicylic acid, 2.0 g phenol, 0.50 g sodium sulfate, and 200.0 g potassium sodium tartrate and brought to a final volume of 1.0 L with distilled H_2_O. Briefly, 100.0 μg/mL of extract was mixed with DNSA (1.0 L) and H_2_O and incubated for 15 min at 100 °C and then read at 575 nm. The sugar content in the extract is expressed in mg glucose equivalent, which was used as the standard.

### 2.4. Assay to Analyze Potential Antioxidant Activity of MA

The antioxidant activity of the extract was initially evaluated using antioxidant abiotic assays. Specifically, the assays performed include ferric reducing power (FRAP), 2,2-diphenyl-1-picryl-drazil (DPPH), elimination of the superoxide anion and hydroxyl radical, and the chelating capacity of iron.

#### 2.4.1. FRAP Assay

The FRAP assay was performed following the protocol previously reported by Benzie et al. [25]. Briefly, the FRAP working solution was prepared by mixing a solution of FeCl_3_ (20 mM), 2,4,6-Tris(2-pyridyl)-S-triazine (TPTZ) and acetate buffer (300.0 mM, pH 3.6). Next, we incubated the extract (25.0–50.0–100.0 μg/mL) with 1.5 mL of working solution for 4 min and measured the absorbance at 595 nm with a BECKMAN DU 640 spectrophotometer (Harbor Boulevard, Fullerton, CA, USA). Blank is represented by the working solution. The results indicate the percentage inhibition of radical activity compared to the blank.

#### 2.4.2. DPPH Assay

The DPPH assay was performed as reported in the literature by Blois, with minor modifications [26]. Briefly, DPPH solution (80.0 μM) was prepared by solubilizing 2,2-diphenyl-1-picrylhydrazyl (DPPH) in methanol. Then, the extract (25.0–50.0–100.0 μg/mL) was mixed with the DPPH solution for 30 min at room temperature and read by a spectrophotometer at 517 nm. The reduction in absorbance value compared with the blank (DPPH solution) indicates the antioxidant capacity of the extract. The antioxidant activity, expressed as % (100% equivalent to the maximum concentration of DPPH, representing the control), was calculated by the following formula:

$$I\left(\%\right)=\left[\frac{Ac-As}{Ac}\right]\times 100$$

Ac is the absorbance of the control and As is the absorbance of the sample.

#### 2.4.3. Superoxide Anion Assay

The superoxide anion elimination assay was performed as reported by Halliwell et al. [27]. Briefly, the extract (25.0–50.0–100.0 μg/mL) was incubated for 5 min, at 25 °C with Nitro blue tetrazolium chloride (NBT) (499.0 mM), NADH (187.0 mM) and phenazine methasulfate 0.600 mM. Then, after the incubation was finished, the solutions were read in the spectrophotometer at 560 nm and the ability of the extract to inhibit anion production was evaluated. Blank is represented by the working solution containing Nitro blue tetrazolium chloride (NBT) (499.0 μM), NADH (187.0 μM) and phenazine methasulfate 0.600 μM. Results are expressed as a percentage, assuming the maximum concentration of superoxide anions as 100% (control).

#### 2.4.4. Hydroxyl Radical Assay

The action of the extract toward hydroxyl radical elimination was evaluated as reported in the literature by Halliwell et al. [27]. Briefly, the extract (25.0–50.0–100.0 μg/mL) was incubated for 30 min with a solution containing 0.025 mM FeCl_3_, 0.104 mM EDTA, 0.10 mM ascorbic acid, and 2.8 mM H_2_O_2_. After incubation, the samples were treated with thiobarbituric acid (1%) and trichloroacetic acid (15%) and placed at 100 °C for 5 min. Subsequently, the samples were cooled and read at the spectrophotometer at 532 nm. Results expressed in % indicate the percentage of radicals present in solution after treatment with the extract. Blank is represented by the working solution containing 0.025 mM FeCl_3_, 0.104 mM EDTA, 0.10 mM ascorbic acid, and 2.8 mM H_2_O_2_. The results are expressed in %, and 100%, which is equivalent to the maximum concentration of Hydroxyl radical, represented by the control.

#### 2.4.5. Iron Chelating Assay

The chelating action of the extract on Fe^2+^ has been evaluated by the method of Decker et al. [28]. Briefly, the extract (25.0–50.0–100.0 μg/mL) was mixed with H_2_O and with FeCl_2_ (2.0 mM) and 3-(2-pyridyl)-5,6-bis(4-phenyl-sulfonic acid)-1, 2, 4-triazine (ferrozine) (5.0 mM) for 20 min. Next, the absorbance was read by a spectrophotometer at 562 nm. The results expressed as percentages indicate the iron present in solution. Blank is represented by the working solution containing H_2_O mixed with FeCl_2_ (2.0 mM) and ferrozine (5.0 mM). The results are expressed in percentages, and 100%, which is equivalent to the maximum concentration of Free Iron, is represented by the control.

### 2.5. Analysis of the Biological Potential MA on RBCs

The biochemical picture of the influence of MA extract on erythrocytes has been made by the evaluation of the degree of hemolysis, chloride/bicarbonate exchange across the cell membrane, its influence on phosphorylation processes, analysis of the oxidative stress levels, morphological changes and activation of apoptotic events.

#### 2.5.1. Preparation of Human Red Blood Cells

Blood samples, stored with EDTA, were washed three times with NaCl 166 mM and treated as previously reported by Tellone et al. [29]. The samples were centrifuged, with a J2-HS Centrifuge, Beckman (Harbor Boulevard, Fullerton, CA, USA), at 3000 rpm, for 5 min at 4 °C; the RBCs were resuspended in buffer (2-[4-(2-hydroxyethyl) piperazin-1-yl]ethanesulfonic acid (HEPES)) at pH 7,40, 20.0 mOsm/Kg, measured by an Omostat OM-6020 (Daiichikagakuco, Kyoto, Japan). A stock erythrocyte solution with a 3% hematocrit was prepared and used for all experiments.

#### 2.5.2. Hemolysis Percentage and Methemoglobin Calculation

The percentage of hemolysis and methemoglobin formed during incubation with the extract was calculated as reported in the literature by Tellone et al. [29]. RBCs (3% hematocrit) were incubated in the presence or absence of MA leaves extract (50.0 and 100.0 µg/mL). Following washing, the supernatant obtained from red blood cells, in the presence and absence of extract (50.0 and 100.0 μg/mL), was used for the calculation of hemolysis. The analysis was carried out through a spectrophotometric reading at 576 nm. The percentage of hemolysis was calculated using the following formula:

H(%)=A/B×100%

where H (%) indicates the percentage of hemolysis occurred, A is the amount of Hb released by the samples and B indicates the maximum Hb released after inducing hemolysis with ultrapure H_2_O.

The calculation of methemoglobin was instead conducted through the method of W.G. Zijlstra et al. [30]. All experimental conditions show meta-Hb and hemolysis values of less than 3%.

#### 2.5.3. Heat-Induced Hemolysis

Following washing, the RBCs (3% hematocrit) were pretreated with MA leaves extract (50.0 and 100.0 μg/mL), with 3% hematocrit, for 30 min. Subsequently, the samples were incubated at different temperatures (37 °C, 45 °C, 50 °C, and 55 °C) for 30 min. At the end of the incubation time, the samples were centrifuged at 3000 rpm for 5 min and the supernatant was read by spectrophotometric analysis at 576 nm. Total hemolysis was assessed by lysis of red blood cells with H_2_O [31].

#### 2.5.4. Modulation of *Morus alba* Extract on Osmotic Fragility

Washed RBCs with 10% hematocrit were treated with the extract (100.0 μg/mL) for 5 and 90 min at 37 °C in HEPES buffer (pH 7.40). Concentrations of 0.34; 0.36; 0.38; 0.40; 0.42; 0.44% were prepared, diluting 50.0 μL of the solution in 1.0 mL of NaCl; 50 μL of RBCs (10% hematocrit) in 950 μL of H_2_O were prepared to obtain 100% lysis and 950 μL of NaCl 0.9% was used as a control to determine the baseline for no lysis. All samples were centrifuged at 3000 rpm for 5 min at a temperature of 4 °C. The supernatant absorbance was recorded at a wavelength of 576 nm [32].

#### 2.5.5. Effect of Hydrogen Peroxide Exposure on Red Blood Cell Membrane Integrity

RBCs (3% hematocrit) suspended in HEPES buffer, pH 7.40, were incubated with the MA leaves extract (100.0 μg/mL) for 30 min at 37 °C. At the end of the incubation time, the samples were centrifuged at 3000 rpm for 5 min. The precipitate obtained was resuspended in a HEPES buffer and treated with hydrogen peroxide (H_2_O_2_) (300.0 mM) for 2 and 14 h. Subsequently, after centrifugation, the percentage of hemolysis was measured in the supernatant derived from each sample. The control sample was prepared by diluting RBCs in HEPES buffer [33]. The hemolysis percentage was assessed by spectrophotometric analysis at 576 nm.

#### 2.5.6. Morphological Analysis of the Red Blood Cell Membrane

Washed RBCs (3% hematocrit) were suspended in a HEPES buffer at 7.40 pH, and incubated with MA leaves extract (100.0 μg/mL), for 30 min at 37 °C. The samples were then centrifuged for 5 min at 3000 rpm and the pellet suspended in a buffer solution was treated with H_2_O_2_ (50.0 mM) for 6 h at 37 °C. After the incubation time, the samples were centrifuged and the pellet diluted 1:1 in Hepes buffer with a hematocrit at 1.5%, was used for morphological analysis, visualized using a Zoe Fluorescent Cell Imager (Bio-Rad Laboratories, Hercules, CA, USA) [33].

#### 2.5.7. Flow Cytometry Analysis

FACS Canto II cytofluorometer (Becton Dickinson, Franklin Lakes, NJ, USA; BD FACSDiva™ software v8.0.3) was used for cytofluorimetric analysis as reported by Carelli-Alinovi et al. [34]. Samples were analyzed by physical gate side scatter (SS) versus forward scatter (FS) using a logarithmic scale. Readings were taken by histogram representation (using a linear scale) and/or by two-dimensional graph representation (cytogram dot blot). Specifically, RBCs with a hematocrit of 3% (three biological replicates (*N* = 3)) were treated for 14 h with MA leaf extract (100.0 μg/mL). Next, samples were washed and diluted 1:5 with a buffer solution at 7.4 pH, containing 0.14 M NaCl, 0.01 M HEPES-NaOH, 2.5 mM CaCl_2_. Annexin V (20 μL of stock solution 1 mg/mL concentration) was then added to each sample, which was incubated in the dark for 15 min and analyzed by flow cytometry. Annexin fluorescence was measured using the FL-1 fluorescence channel, by examining the direct scatter analysis. The excitation and emission wavelengths were 488 nm and 530 nm, respectively.

#### 2.5.8. Kinetic Measurements

AE1 can exchange different anions at different rates, and using sulfate ions to monitor transport activity gives the advantage of a slower exchange time. This allows for a relatively simple experimental process to be organized. In addition, the virtual absence of sulfate ions within the erythrocyte ensures that intracellular sulfate determinations are essentially indicative of transport [35].

RBCs were buffered (35.0 mM Na_2_SO_4_, 90.0 mM NaCl, 25.0 mM HEPES buffer and 1.5 mM MgCl_2_), at 25 °C, pH 7.5, in the presence and absence of the extract (50.0 and 100.0 μg/mL) [36]. Subsequently, at different time intervals (5, 15, 30, 60, 90 and 120 min), 10.0 mM of 4-acetamide-40-isothiocyanostilbene-2,20-disulfonic acid (SITS) was added to stop the reaction. Then, through three washes at 3000 rpm for 5 min at 4 °C, the incubation buffer and the sulfate that did not enter the cells were removed. Following the last wash, the RBCs were treated with perchloric acid (4%) and distilled H_2_O and centrifuged for 10 min at 10,000 rpm. We added a mixture of glycerol and distilled water (1:1, *v*/*v*), NaCl 4 M, 1 M HCl and BaCl_2_∙2H_2_O 1.23 M to remove sulfate ions from the supernatant and obtain a homogeneous precipitate of barium sulfate. The absorbance of the mixture was measured between 350 and 425 nm. The concentration of sulfate was measured with a calibrated standard curve, established by measuring the absorbance of suspensions containing known amounts of sulfate [35]. The results were analyzed through the following equation:

c(t)=c∞(1 e kt)

where c(t) indicates the concentration of sulfate at time t, c∞ indicates the intracellular concentration of sulfate at equilibrium, and k is the rate constant of the sulfate influx.

#### 2.5.9. ATP Measurement

ATP values, intra and extracellular, were evaluated by the luciferin-luciferase method [37]. The value of light released is directly proportional to the concentration of ATP contained within the samples. Briefly the erythrocytes (3% hematocrit) treated or not with MA leaves extract (50.0 and 100.0 μg/mL final concentration) for 30 min, were diluted and incubated for one hour with Mastoparan 7 (Mas 7), an inducer of Gi proteins. After incubation with Mastoparan, RBCs were centrifuged 3 times, at 3000 rpm at 4 °C for 5 min. The obtained supernatant was stored to calculate the extracellular ATP value. The pellet was deproteinized with trichloroacetic acid (TCA), washed again and after the last centrifuge (at 3000 rpm at 4 °C for 5 min) a solution of D-luciferin and Firefly lantern extract (FLE 250) was added to the samples at a ratio of 1:1. The light emitted was recorded using a Bio Orbit 1251 luminometer (Bio-Orbit Oy, 297 Turku, Finland) and represents intracellular ATP [38,39]. A calibration curve for ATP determination was prepared using standard (ATP) at different concentrations (from 0 to 10^−9^ M). ATP assay mixture (containing luciferase enzyme), purchased from Sigma Aldrich, was then added to the solutions and the samples were read on a luminometer.

#### 2.5.10. Determination of Phosphatase PTP-1B Activity

PTP-1B activity was measured by the assay described above by Maccaglia et al. with minor modifications [40]. Briefly, the RBCs membranes, in the presence and absence of MA leaves extract (50.0 and 100.0 μg/mL final concentration), were compared with the results obtained from samples treated with orthovanadate (OV) (3.0 mM), phosphatase inhibitor and using p-nitrophenyl phosphate (p-NPP) as a substrate. The membranes were resuspended in HEPES buffer (25.0 mM) containing 0.1 mM phenylmethanesulfonylfluoride (PMSF), 20 mM MgCl_2_ and 15 mMp-NPP and incubated at 37 °C for 60 min. Subsequently, the samples were centrifuged, and the release of p-nitrophenol was detected at 410 nm [40].

#### 2.5.11. Caspase 3 Assay

Washed RBCs (3% hematocrit) suspended in HEPES buffer (pH 7.40) were incubated in the presence and absence of the MA leaves extract (50.0 and 100.0 μg/mL) and tert-butyl-hydroperoxide (t-BHT) 100.0 mM, a well-known oxidant and caspase for 1 h at 37 °C [41,42]. RBCs were then centrifuged at 3000 rpm for 5 min, and the pellets were lysed by sonication. Each sample was centrifuged for 10 min at 15,000 rpm, and the supernatant was filtered through a Microcon YM 30 (nominal molecular weight limit 30.000) to obtain purified caspase. Finally, the samples were incubated with the specific substrate of caspase 3 AcDEVD-pNA, at 37 °C for 1 h. The release of p-nitroaniline (pNA) is being evaluated using spectrophotometric analysis at a wavelength of 405 nm.

#### 2.5.12. Determination of Sulfhydryl Groups

Analysis was performed according to the method of Aksenov et al. [43]. Following washes, erythrocytes (3% hematocrit) were suspended in HEPES buffer, pH 7.40, with a. Subsequently, treated and untreated samples with the MA leaves extract (50.0–100.0 μg/mL) were incubated with 2,2-Azobis(2-methylpropionamidine) dihydrochloride (AAPH) 100.0 mM for 1 h at 37 °C. After incubation, the samples were centrifuged for 5 min at 3000 rpm, the supernatant discarded, and the pellet lysed with cold H_2_O (1:1). Next, 50.0 μL was taken and made up to 1.0 mL with PBS, pH 7.40, containing EDTA (1.0 mM). Then the samples were incubated with 5,5′-Dithiobis (2-nitrobenzoic acid) (DTNB) 10.0 mM, for 30 min at 25 °C, in the dark. After incubation, absorbance was read at 412 nm. The data, expressed as %, indicates the percent reduction of total thiol groups present in the samples (100% is represented by control).

#### 2.5.13. Lipid Peroxidation

Lipid peroxidation levels were assessed as reported in the literature by Mendanha et al. [44]. The erythrocytes (3% hematocrit), after washing, were treated with MA leaves extract (50.0–100.0 μg/mL) and incubated with 100.0 mM AAPH for 1 h at 37 °C. Subsequently, the samples were centrifuged at 3000 rpm for 5 min, suspended in lysis buffer (Tris 5 mM, KCl 5 mM, pH 7.40) and treated with 15% trichloroacetic acid (TCA). Subsequently, the samples were centrifuged at 10,000 rpm for 5 min, the precipitate discarded, and the supernatant was removed, mixed with thiobarbituric acid (TBA) (1%) and incubated at 100 °C for 30 min. After incubation, the samples were cooled and read at 532 nm. Results are expressed as %, and 100% is represented by RBCs treated with AAPH (100.0 mM)

### 2.6. Statistical Analysis

All results are an average of three biological replicates (*N* = 3) and three technical replicates (*n* = 3). The average of the technical replicates was calculated within each biological replicate, and then these averages were used to calculate the final mean utilized for statistical analysis. The data are expressed as mean ± standard error of the means (SEM) and were statistically evaluated for differences using one-way analysis of variance (ANOVA), followed by the Tukey–Kramer test (SigmaPlot Version 12.0, Systat Software Inc., San Jose, CA, USA and GraphPad Software, version 8.0, San Diego, CA, USA). *p*-values less than or equal to 0.05 were considered significant.

## 3. Results

The first step in our study was to evaluate the cytotoxicity of the tested concentration utilized during our experimentation by hemolysis and methemoglobin formation. The results obtained showed no significant statistical difference between the control and the RBCs incubated with 50.0 and 100.0 μg/mL of MA extract (Supplementary Materials, Figures S1 and S2 and Table S1). Then we evaluate the phenolic, flavonoid and carbohydrate content of the dry leaf ethanolic extract. Phytochemical analyses, already available in the literature, highlight that the MA leaf extract contains different molecules, including coumarins, tannins, triterpenes, flavonoids, cinnamic acids, alkaloids, neochlorogenic acid and its isoforms, carbohydrates, caffeic acid and phenolic compounds. The data in the table (Table 1) shows a similar content of phenols and flavonoids in our extract; total phenolic content checked by gallic acid was about 2.140 mg gallic acid equivalents, and the value of total flavonoids checked by quercetin was about 2.200 mg quercetin equivalents in 100.0 μg/mL of extract, respectively. Total carbohydrate content expressed in mg of glucose equivalents was small compared to phenols and flavonoids, being a value of about 0.970 mg in 100.0 μg/mL of extract.

Phenolic compounds are well-known for their strong antioxidant properties due to the aromatic ring in their chemical structure, especially the location and number of hydroxyl groups. They can neutralize free radicals and reduce the harmful effects of oxygen species.

### 3.1. Antioxidant Activity of the Extract

**Figure 1:**
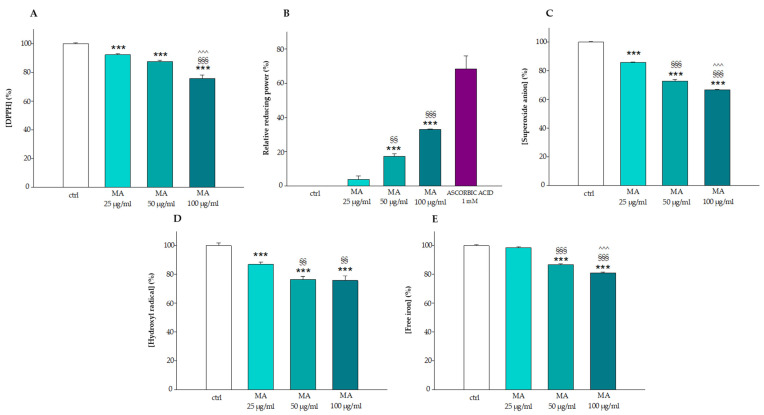
The antioxidant action of *MA* leaf extract at different concentrations (25.0, 50.0, 100.0 μg/mL) was evaluated by the most common antioxidant assays such as DPPH (Section (**A**)), FRAP (Section (**B**)), superoxide ion elimination assay (Section (**C**)), hydroxyl radical elimination assay (Section (**D**)) and iron chelation assay (Section (**E**)). *** *p* < 0.001 vs. ctrl, §§ *p* < 0.01 vs. 25.0 μg/mL, §§§ *p* < 0.001 vs. 25.0 μg/mL, ^^^ *p* < 0.001 vs. 50.0 μg/mL. Statistical analysis performed by one-way ANOVA followed by Tukey’s test (*n* = 3).

Erythrocytes, due to their functional activities of oxygen transport, are highly exposed to oxidative damage. In this context, the ability of the extract (25.0–50.0–100.0 μg/mL) to neutralize and counteract free radicals was evaluated by different abiotic antioxidant assays, such as DPPH, FRAP, superoxide anion and hydroxyl radical elimination assay, and iron chelation assay (see Figure 1). Specifically, DPPH is a nitrogenous free radical with a strong absorption at 517 nm, being efficiently scavenged by antioxidants. Its neutralization can be considered representative of the assay of the scavenger activity of a compound. As shown in Section A of Figure 1, the generation of DPPH radicals is gradually reduced by MA extract, reaching a decrease of about 25% at the highest concentration of MA tested. Section B shows the ferric reducing ability of MA extract, based on the reduction of ferric-tripyridyltriazine to ferrous-tripyridyltriazine complex with an absorption maximum at about 595 nm. The rate of ferric reducing ability by the extract increased in a concentration-dependent manner in the concentration range of 25.0 to 100.0 mg/mL; in detail, 100.0 μg/mL of MA extract shows the highest antioxidant capacity (about 35%) compared to 50.0 μg/mL (about 18%) and 25 μg/mL (about 5%). Sections C and D of Figure 1 reflect the free radical scavenger strength of MA extract for superoxide anion and hydroxyl radical, respectively; the scavenging activity against superoxide anion increased in a concentration-dependent manner within the range of 25.0–100.0 μg/mL with the maximum effect being reached at the concentration of 100.0 μg/mL (about 40%) (see Section C). The ability to neutralize the hydroxyl radical already detectable at 25.0 μg/mL increased in a concentration-dependent manner to 50.0 μg/mL, which in this case, is the plateau of antioxidant activity (about 30%) (see Section D). Finally, MA extract (100.0 μg/mL) shows an iron chelation capacity of about 20%, but the effect was not observed before 50.0 μg/mL (see Section E).

The generation of free radicals is dangerous for cells because their interaction with membrane phospholipids causes lipid peroxidation, which can destroy the integrity of the cell membrane. The protective capacity of the extract was tested on human RBCs, evaluating its impact on membrane integrity.

### 3.2. Regulation of Osmotic Fragility by Morus alba Extract

**Figure 2:**
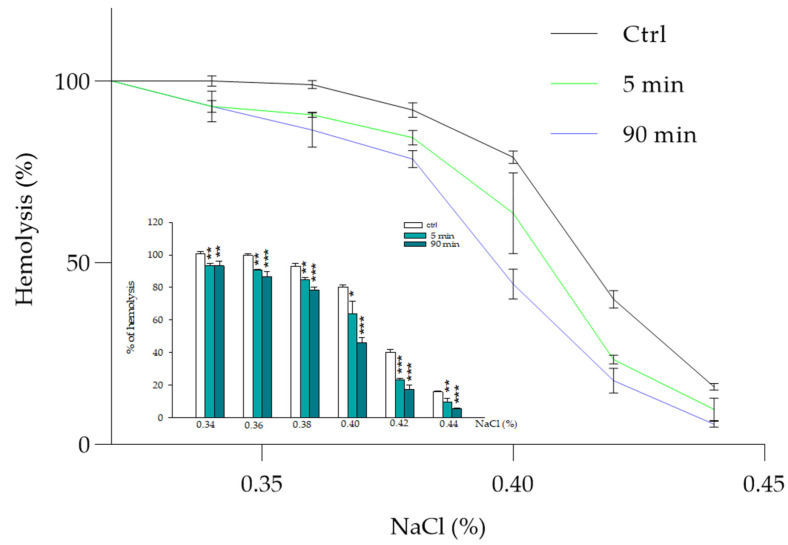
Protective effect of MA extract 100.0 μg/mL on erythrocyte membrane fragility after incubation with different NaCl concentrations. The percentage of hemolysis, measured by spectrophotometric abs at 576 nm, is plotted as a function of the percentage of sodium chloride concentration (NaCl %). The percentage of hemolysis is evaluated from the abs measurement obtained after incubation with pure water (100% hemolysis). * *p* < 0.05 vs. ctrl, ** *p* < 0.01 vs. ctrl, *** *p* < 0.001 vs. ctrl. Statistical analysis performed by one-way ANOVA followed by Tukey’s test (*n* = 3).

Membrane elasticity and deformability are essential for RBCs’ survival and for effective blood flow. Changes in osmolarity can interfere with the shape and volume of RBCs, as observed by Ponder, erythrocytes in hypotonic solutions tend to swell due to an imbalance in solute concentration that causes an increase in H_2_O in the cellular environment, leading to cell lysis [45]. Figure 2 shows the effect of changes in osmolarity on the erythrocyte solution incubated with MA extract (100.0 μg/mL) in the range of concentration of NaCl that induces initial hemolysis (0.45%), or complete hemolysis (0.35%) as reported by Durgawale et al. [46]. The RBC hemolysis percentage decreases physiologically as NaCl increases, but the effect of treatment with MA extract is detectable throughout all the tested ranges, both for 5 and 90 min of incubation time. The maximum protective effect was reached between 0.40 and 0.42 NaCl (%), both after 5 and 90 min. Specifically, incubation of RBCs with the extract, for 5 min, shows a reduction in hemolysis of about 15 and 20%, compared with the control (0.40 and 0.42% NaCl, respectively), a value that increases when 90 min of incubation is reached (reduction of about 30 and 25%, respectively).

### 3.3. Heat-Induced Hemolysis

**Figure 3:**
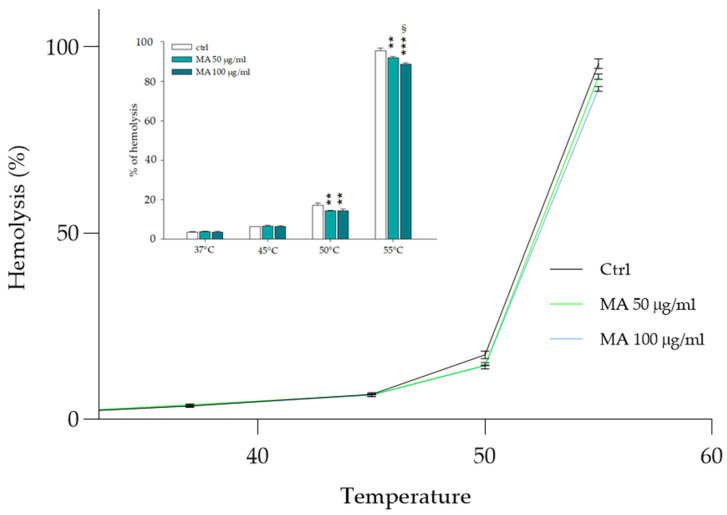
Protective effect of MA extract on the integrity of the erythrocyte membrane as the temperature increases. RBCs pretreated with MA extract (50.0 and 100.0 μg/mL) were incubated for 30 min at temperatures of 37°, 45°, 50° and 55 °C. The hemolysis percentage, derived by spectrophotometric measures at 576 nm, is plotted in relation to temperature increase. ** *p* < 0.01 vs. ctrl, *** *p* < 0.001 vs. ctrl, § *p* < 0.05 vs. 50.0 μg/mL. Statistical analysis performed by one-way ANOVA followed by Tukey’s test (*n* = 3).

The movements of erythrocytes through the vascular system into the bloodstream are related to the ability of these cells to deform, adapting to different vessels sizes. In addition to osmolarity, the erythrocyte membrane deformability is related to its chemical composition, and it is strictly dependent on different physiological and non-physiological factors, which could lead to stiffening and consequently to the rupture of the same. Among the different factors involved in the destabilization of membrane elasticity is the temperature of the solution in which the RBCs are suspended. RBCs’ effective stiffness decreases when temperature increases due to the lipid tails that become less ordered, allowing them to move more freely within the bilayer [47]. In Figure 3, the effect of the MA extract is shown on different temperature solutions of RBCs. The cells were pretreated with MA extract (50 and 100.0 μg/mL) and incubated for 30 min at temperatures from 37 °C to 55 °C. The tested temperature range was selected to encompass the physiological temperature at which RBCs normally work (37 °C) up to the value at which they move to spherocytes, show budding, and fragmentation (50–55 °C) [48]. Interestingly, a slight but significant protective effect of the extract becomes evident when the temperature exceeds 45 °C.

The protection exerted against damage caused by exposure to high temperatures suggests the accumulation of MA extract in the membrane phospholipid bilayer. In this case, the presence of the extract in the RBC membrane could help to maintain membrane fluidity and stability, which is crucial for protecting the cell from environmental changes, including high temperatures.

### 3.4. Effect of Hydrogen Peroxide on Erythrocyte Membrane

**Figure 4:**
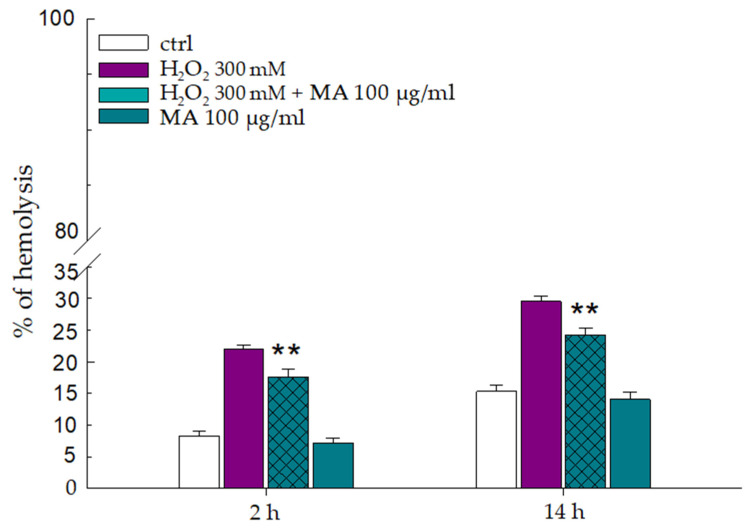
Inhibitory ability of MA extract on the RBCs hemolysis induced by H_2_O_2_ exposition. Absorbance measured at 576 nm is plotted in relation to the incubation time of RBCs with H_2_O_2_. RBCs in the absence of both MA extract and H_2_O_2_ (ctrl), RBCs exposed only to H_2_O_2_ (300.0 mM), RBCs pretreated with MA extract (100.0 μg/mL) for 30 min and exposed to H_2_O_2_ (300.0 mM), RBCs with MA extract (100.0 μg/mL). ** *p* < 0.001 vs. ctrl. Statistical analysis performed by one-way ANOVA followed by Tukey’s test (*n* = 3).

Hydrogen peroxide is a strong oxidizing agent; its interaction with phospholipids triggers lipid peroxidation, which can destroy the integrity of the cell membrane, leading to the hemolysis of RBCs. The potential of MA extract to protect against oxidative damage caused by hydrogen peroxide was also evaluated. With this aim, RBCs pretreated for 30 min with MA extract (100.0 μg/mL) were incubated with H_2_O_2_ (300.0 mM) for 2 and 14 h, respectively. As shown in Figure 4, RBCs incubated in the absence of H_2_O_2_ were stable, with limited hemolysis observed. In contrast, RBCs incubated with H_2_O_2_ (300.0 mM) were oxidatively damaged, with hemolysis rates of about 20 and 25% after exposure of 2 and 14 h, respectively. After the pretreatment of RBCs with MA extract (100.0 μg/mL), the hemolysis inhibition rate was about 20% both at 2 and 14 h. Interestingly, although there was a change in hemolysis percentage from 2 h and 14 h in each sample, the inhibitory power exerted by MA does not change even by increasing the exposure time (14 h) to H_2_O_2_.

### 3.5. Effect of Morus alba on RBCs Morphology

**Figure 5:**
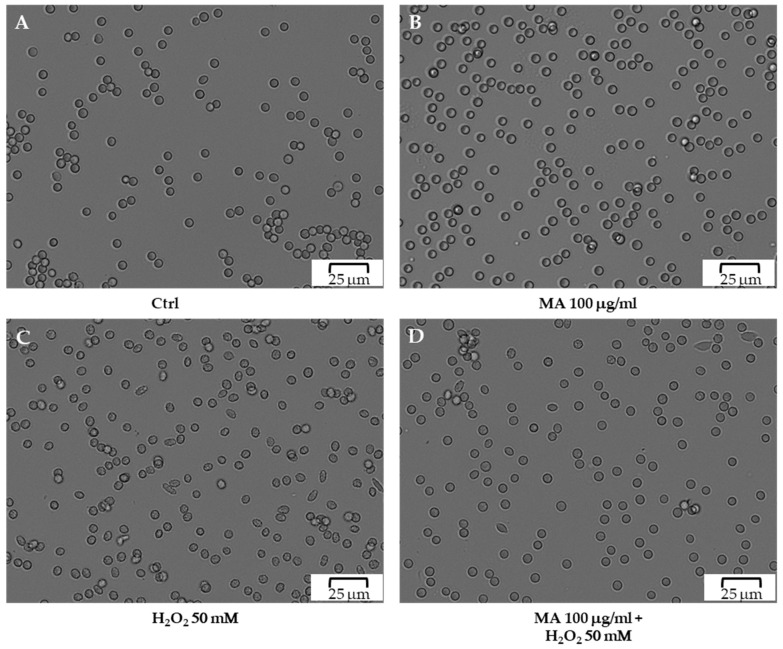
Images of control RBCs (Ctrl) and after 6 h of incubation with MA extract (100.0 μg/mL) or H_2_O_2_ (50.0 mM), respectively. Bottom right, the image of RBCs pretreated with MA extract (100.0 μg/mL) for 30 min and incubated for 6 h with H_2_O_2_ (50.0 mM). In detail section (**A**) represents control (untreated RBCs); section (**B**) represents RBCs treated with the extract (100.0 μg/mL); section (**C**) represents RBCs treated with H_2_O_2_ (50 mM); section (**D**) shows RBCs treated with the extract and exposed to the oxidant (H_2_O_2_). Scale bars 25 μm.

To better characterize the damage to RBCs caused by hydrogen peroxide and the protection of the MA extract, cell morphology was examined. Figure 5 shows the RBCs morphology evaluated in normal conditions (A), after 6 h of incubation of RBCs with MA extract (B) or H_2_O_2_ (C), and, in RBCs pretreated with MA extract for 30 min and after incubating for 6 h with H_2_O_2_ (D). On the left side of the image (A), it is possible to observe the classic biconcave disc shape typical of the normal RBCs (about 1–2% of echinocytes) that is largely maintained in the presence of MA extract (about 10–15% of echinocytes) (B). The right side of the image shows the shrinkage and alteration of the erythrocyte structure due to the action of the oxidant (C) and the partial restoration when RBCs are pretreated with the extract (D). Therefore, the results show that morphological alterations highlighted by spicules (about 80–90% compared to the control) protruding from the surface of the cell membrane, induced by the oxidant (C) are partially inhibited (about 50% of inhibition) by the presence of MA (D).

The morphological alterations on RBC induced by hydrogen peroxide and their partial inhibition by the MA extract are in line with the above analyses and confirm the beneficial action on the cell by the extract. To further study the MA impact on the RBCs functionality, the kinetics of anion exchange between the inside and outside of the cell were evaluated.

### 3.6. Influence of Morus alba on Anion Exchange

**Figure 6:**
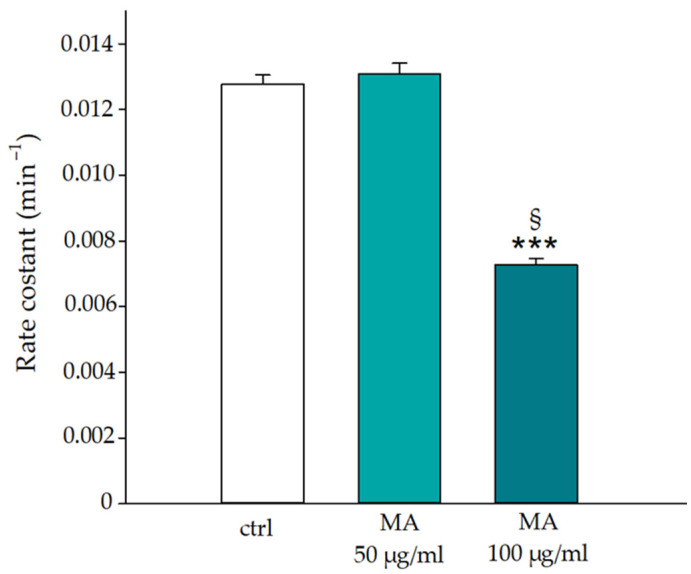
Effects of MA extract on rates of sulphate transport in normal human RBCs, incubated in the absence (ctrl) or in the presence of the extract (50.0 and 100.0 μg/mL). *** *p* < 0.001 vs. ctrl, § *p* < 0.05 vs. 50.0 μg/mL. Statistical analysis performed by one-way ANOVA followed by Tukey’s test (*n* = 3).

Anion exchanger 1 (AE1), also known as Band 3 protein, is a member of the solute transporter family (SLC4A1). AE1 is the most abundant protein in the erythrocyte membrane, where it is responsible for the electroneutral chloride/bicarbonate anion exchange. Its structural integrity is critical for cellular viability and efficiency by acting as an anchor for the cytoskeleton and playing a role in senescence cell antigen. The influence of the extract on AE1 functionality, studied through a spectrophotometric evaluation of the anion exchange kinetic values, shows an inhibition of the sulphate flux rate of approximately 60% (see Figure 6). Specifically, the kinetics were tested at two concentrations of the extract 50.0 μg/mL and 100.0 μg/mL, comparing the results with the control, i.e., RBCs in the absence of MA extract. The value of the rate constant for the kinetics recorded on RBCs in the absence of the extract (ctrl) is 0.012 min^−1^, a value similar to that calculated on erythrocytes treated with 50.0 μg/mL of the extract equal to 0.013 min^−1^, demonstrating that this concentration does not affect the anion flux as there are no significant variations compared to the control sample. It is different from what happens at higher concentrations of extract (100.0 μg/mL), in which the variation in the rate constant (from 0.012 to 0.0071 min^−1^) compared to the control sample shows a decrease in the anion exchange rate of about 60%.

This decrease in anion exchange can be dangerous for the RBCs’ viability because AE1 functionality is essential for membrane stability and cell shape. In addition, a reduced exchange of ${HCO}_{3}^{-}/{Cl}^{-}$ slows down CO_2_ elimination, causing a dangerous generation of free radical species.

### 3.7. Oxidative Status of RBCs

**Figure 7:**
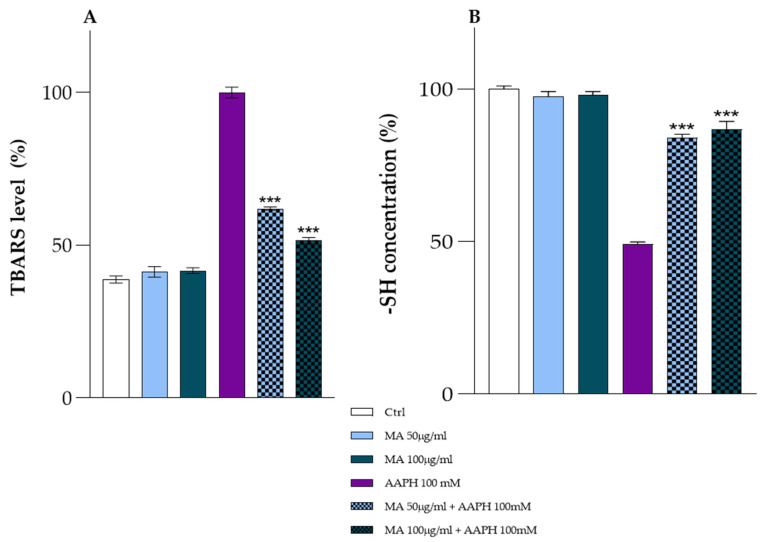
TBARS levels (%) (Section (**A**)) and total sulfhydryl group content (%) (Section (**B**)) measured in RBCs in the absence of the MA extract (ctrl), RBCs incubated for 1 h with AAPH (100.0 mM), RBCs incubated for 1 h with MA extract 50.0 or 100.0 μg/mL, RBCs pretreated with MA extract 50.0 μg/mL after incubated with AAPH (100.0 mM), RBCs pretreated with MA extract 100.0 μg/mL after incubated with AAPH (100.0 mM). *** *p* < 0.001 vs. AAPH 100.0 mM. Statistical analysis performed by one-way ANOVA followed by Tukey’s test (*n* = 3).

The alteration of the anion flux recorded in the presence of MA extract could have effects on the Hb functionality and oxidative state of the cell. Our results exclude this hypothesis. At all the concentrations tested, the percentages of methemoglobin always remain lower than 3%. The oxidative status of RBCs was assessed by measuring thiobarbituric acid reactive substance (TBARS) levels and total thiol groups (see Figure 7). TBARS in RBCs is one of the most common markers of oxidative stress, derived from lipid peroxidation. The technique is based on the reaction with malondialdehyde (MDA), one of the components commonly measured by TBARS. Section A of Figure 7 shows no alteration in TBARS levels in RBCs treated for 1 h with MA extract (50.0 and 100.0 μg/mL) compared to RBCs in the absence of the extract (ctrl). These results, in line with our previous data showing no oxidative damage on RBCs exposed to MA extract, support the protective action of the extract highlighted by the last two histograms in Section A (Figure 7). Specifically, histograms show a decrease in TBARS levels of about 40% in RBCs pretreated for 1 h with MA extract (50.0 μg/mL) and approximately 50% in RBCs pretreated for 1 h with MA extract (100.0 μg/mL), compared to cells treated with AAPH for 1 h. The inhibitory action of the extract toward AAPH oxidative damage was also evaluated by assessing the oxidative status of thiol groups. In RBCs, thiol groups are attributed to the thiol group of cysteine b-93 of Hb and to the thiol group of glutathione (GSH), one of the most important endogen antioxidants. Thiol groups are essential for maintaining cell integrity and defending against oxidative stress. As shown in Section B of Figure 7, the extract does not alter the oxidative state of the total thiol groups but rather protects them from potential AAPH-induced oxidation. Notably, RBCs treated for 1 h with AAPH (100.0 mM) show an oxidation of the -SH groups of about 50%, compared with the control (ctrl), a condition that is almost restored in the presence of extracts 50.0 and 100.0 μg/mL (15% of the -SH groups oxidized).

### 3.8. Effect of Morus alba Extract on ATP Levels and PTP-1B Activity

**Figure 8:**
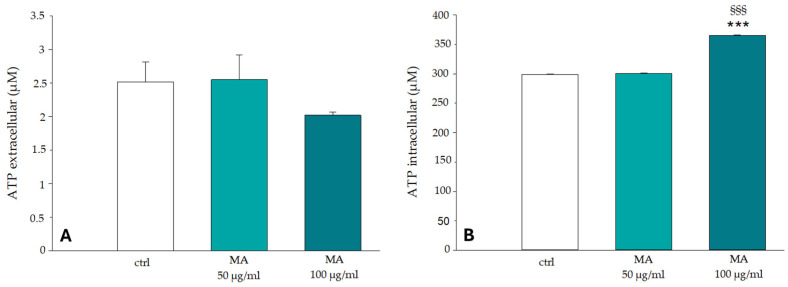
Effect of MA extract on the extracellular (**A**) and intracellular (**B**) ATP levels in RBCs. ATP concentrations were measured at the end of the incubation period of RBCs in the absence (ctrl) or in the presence of 50.0 μg/mL and 100.0 μg/mL extract. *** *p* < 0.001 vs. ctrl, §§§ *p* < 0.001 vs. MA 50.0 μg/mL. Statistical analysis performed by one-way ANOVA followed by Tukey’s test (*n* = 3).

In RBCs, free radical levels and ATP levels are closely related, because in these cells, ATP production is essentially based on the glycolytic pathway. Glycolysis itself is metabolically regulated by the binding of glycolytic enzymes (GE) with the cytoplasmic domain of band 3 protein (CDB3) [49]. The results in Figure 8 show the ATP values recorded in RBCs incubated in the presence and absence of the MA extract; in Section A, extracellular ATP values are displayed, while in Section B, the intracellular ATP values are shown. The results in Figure 8 show the ATP values recorded in RBCs incubated in the presence and absence of the MA extract; in Section A, extracellular ATP values are displayed, while in Section B, the intracellular ATP values are shown. The results in Figure 8 show the ATP values recorded in RBCs incubated in the presence and absence of the MA extract; in Section A, extracellular ATP values are displayed, while in Section B, the intracellular ATP values are shown. RBCs incubated with concentrations of 50.0 μg/mL and 100.0 μg/mL of MA extract do not undergo significant changes in ATP release from cells compared to control samples (Section A); while the treatment with 100.0 μg/mL of MA extract causes an increase in the intracellular concentration of the nucleotide compared to RBCs in the absence of the extract (Section B). Intracellular ATP levels are crucial for maintaining RBCs’ shape and function. Thus, the revealed results are in line with morphological images, where no differences are observed between the control (RBCs not exposed to MA extract) and the RBCs treated with MA.

In fact, if intracellular ATP levels decrease, RBCs change their shape, become stiffer and less deformable, leading to the formation of echinocytes.

**Figure 9:**
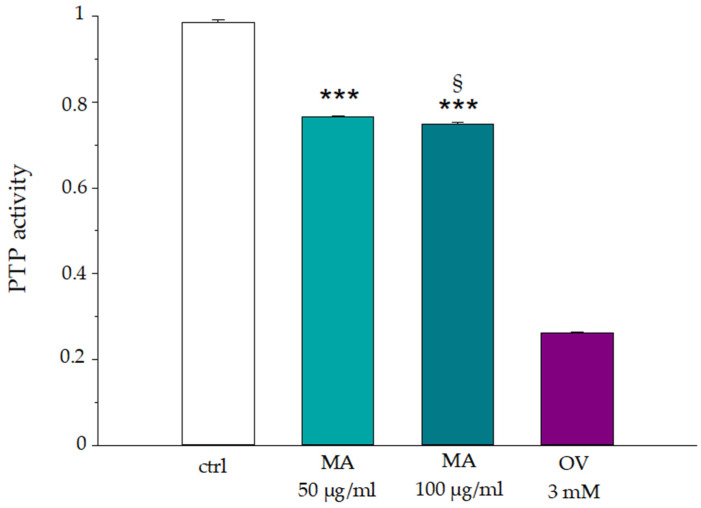
Phosphatase activity in human erythrocytes incubated in the absence (ctrl) or in the presence of MA extract, 50.0 μg/mL, 100.0 μg/mL or Orthovanadate 3.0 mM. *** *p* < 0.001 vs. ctrl, § *p* < 0.05 vs. MA 50.0 μg/mL. Statistical analysis performed by one-way ANOVA followed by Tukey’s test (*n* = 3).

The slight increase in intracellular ATP concentrations may also be correlated with an alteration of phosphatase activity because studies demonstrate that adenine nucleotide can mediate the activity of PTP-1B, acting as an activator of the protein [50]. To verify this modulation, the activity of PTP-1B was tested in RBCs pretreated for 30 min with MA extract (50.0 μg/mL and 100.0 μg/mL), comparing the results with those obtained in the presence of orthovanadate (3.0 mM), a known phosphatase inhibitor. The results in Figure 9 show a slight inhibition of phosphatase activity already in the presence of 50.0 μg/mL of the extract. However, the effect is not concentration-dependent, because increasing the concentration of the extract to 100.0 μg/mL did not reveal any increase in enzyme inhibition.

PTP-1B inhibition may be the cause of a metabolic alteration triggered by an imbalance of the AE1 phosphorylated state. The increased phosphorylation of AE1 triggers the detachment of GE from CDB3 and their subsequent activation, leading to an increase in glycolytic flux. Metabolic shift to the glycolytic pathway can, in turn, cause a dangerous increase in the oxidative state in the cell. Specifically, oxidative stress can activate pro-apoptotic signaling pathways, leading to the activation of caspase 3 and ultimately to RBCs’ death.

### 3.9. The Influence of Morus alba Extract on Caspase 3 Activity

**Figure 10:**
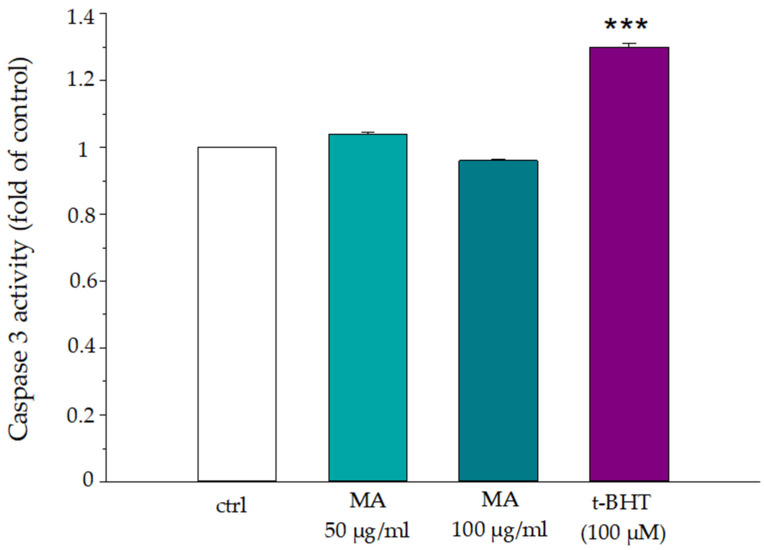
Influence of MA extract on caspase 3 activity in human RBCs. Erythrocytes were treated as described in “Materials and methods”, caspase 3 activity was tested in the absence (white) or in the presence of the extract or t-BHT which represented the positive control. *** *p* < 0.001 vs. ctrl. Statistical analysis performed by one-way ANOVA followed by Tukey’s test (*n* = 3).

Caspase 3 activity has been tested in RBCs incubated with MA extract 50.0 and 100.0 μg/mL for 1 h. Figure 10 shows the results of RBCs in the absence of extract (ctrl), after 1 h of incubation with MA extract (50.0 or 100.0 μg/mL), and after 1 h of incubation with t-BHT a potent inducer of oxidative stress and caspase 3 activation. It is evident that MA extract does not induce caspase 3 activation at either 50.0 or 100.0 μg/mL extract compared to control and t-BHT.

In RBCs, the activation of caspase 3 plays a role in the externalization of phosphatidylserine (PS) and the consequent RBC phagocytosis. Caspase 3 activation destroys the normal lipid arrangement in the cell membrane, leading to PS externalization, which is normally located on the inner leaflet of the membrane. The externalization of PS is a signal to engulf RBCs by macrophages; this recognition is important for the efficient clearance of apoptotic cells.

### 3.10. Flow Cytometry

**Figure 11:**
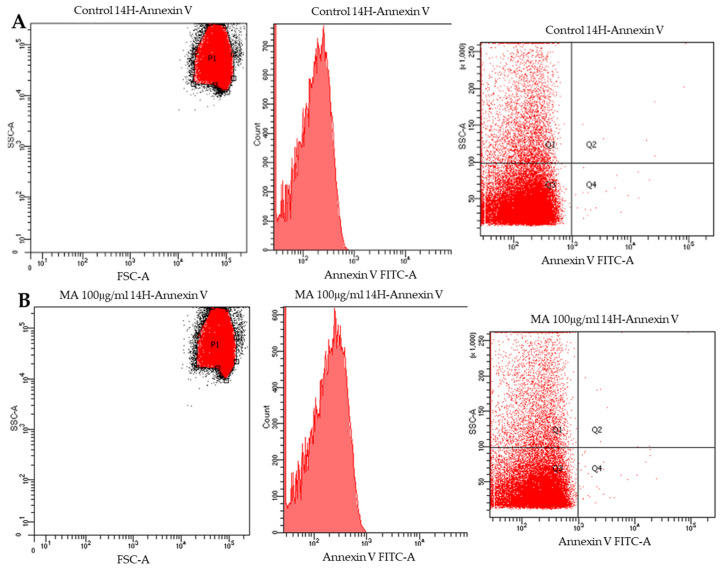
The panel shows the cytofluorimetric analysis of samples incubated in the absence (Section (**A**)) and in the presence of MA extract (100.0 μg/mL), for 14 h (Section (**B**)). Three replicates for each test group (*n* = 3).

To confirm both the lack of caspase 3 activation and apoptotic cell death triggering, cytofluorimetric analysis on RBCs treated with MA extract was performed. The analysis exploits the ability of Annexin V to readily bind PS when exposed to the outer leaflet of the RBC membrane. As shown in Figure 11, the extract (100.0 μg/mL) has a negligible effect on cell viability, does not cause damage to the erythrocyte membrane and does not alter the state of RBC well-being.

## 4. Discussion

Like previous studies, our research results showed a beneficial effect of MA extract expressed as a sort of protection on RBCs from hemolysis induced by various agents, including temperature, osmotic changes and oxidative stress (see Figure 2, Figure 3, Figure 4 and Figure 5) [2,15,20,51]. This protective activity of MA on RBCs, which helps them resist damage caused by the external environment, could be attributed to the formation of an external coating, probably due to the arrangement of the extract in the phospholipid bilayer. Some molecules in the extract, such as polyphenols and carbohydrates, could enhance the RBCs’ resistance to damage and improve overall cell health [52,53]. Polyphenols, acting as powerful antioxidants, can neutralize free radicals, reducing lipid peroxidation and hemolysis.

As shown in the literature, the extract is rich in natural bioactive compounds, such as caffeic acid, rutin, kaempferol, quercetin, chlorogenic acid and DNJ, making it potentially suitable for these functions [4,5,6,7]. Carbohydrates could help to create a protective layer that shields the cell from damage, serving as a protective barrier, contributing to cell membrane integrity. An external interaction could also explain the decrease in the detected anion flux, in this case, a hindrance action may be hypothesized. Like the ATP release from the cell, we also detected a reduced release, which could be affected by the external layering of the extract (see Figure 8). Furthermore, although there are no significant hemolysis values (<3%), a further explanation for the decrease in extracellular ATP values could be attributed to the release, albeit minimal, of enzymes capable of hydrolyzing ATP. However, the action of the extract on intracellular targets also cannot be excluded; this is the case of PTP-1B, whose activity was inhibited in the presence of the extract (see Figure 9).

In this case, the inhibition of PTP-1B could be partly due to the presence of quercetin and kaempferol in the MA extract, as shown by Dugo et al. [4]. Both compounds have a hydroxyl group on the C4 of the B ring that has been shown to enhance the inhibition of PTP-1B by these molecules in comparison with similar compounds [54,55,56]. As already mentioned, the functional inhibition of PTP enzymatic activity causes a phosphorylated imbalance on AE1 that could be responsible for the alteration of the anion exchange in the presence of MA (see Figure 6). AE1 functionality is modulated by several factors, including changes in Hb conformation, interactions with other proteins, the cell’s metabolic state and its phosphorylation state [40,57]. PTP-1B catalyzes the removal of phosphate groups from tyrosine residues on the protein. By dephosphorylating AE1, PTP-1B helps to modulate the functionality and prevent hyperphosphorylation. The protein has, in fact, some amino acid residues that constitute phosphorylation sites, including tyrosine Y8 and Y21 located near the N-terminal domain; Y359 between the membrane and cytoplasmic domains; and Y904 near the C-terminal domain [58,59,60,61]. The phosphorylation of the tyrosine residues of AE1 is synergistically regulated by the activity of tyrosine kinase and phosphatase proteins, p72 (syk), p56/53 (lyn), PTP1B and SHPTP-2 [62]. The hyperphosphorylation of AE1 is associated with an increase in the binding of Hb to CDB3, with the consequent detachment and activation of GE [63,64]. This condition would increase the glycolytic pathway and could be reflected in the revealed increase in intracellular ATP. In fact, although AE1 phosphorylation does not directly generate ATP, it can modulate metabolic pathways that contribute to ATP production [65]. This modulation can affect the overall ATP yield and the cell’s ability to meet its energy demands. However, AE1 hyperphosphorylation and anion exchanger inhibition are two events that can lead RBCs to a variety of adverse effects, including alterations in metabolic and acid-base balance, free radicals’ generation, impaired membrane stability and hemolysis [66,67,68]. A negative metabolic picture tending to cell death, however, was not detected in the presence of MA by our studies. On the contrary, the results of our study are all oriented toward a beneficial and protective action of the extract on the health and vitality of RBCs (see Figure 11). The beneficial effect is most likely due to the presence of bioactive compounds, including quercetin, kaempferol, rutin, caffeic acid, and DNJ. A study conducted by Asgarya et al. showed that rutin, quercetin and kaempferol possess antioxidant properties [69]. Specifically, it has been shown that these compounds inhibit the oxidizing action of AAPH, reducing hemolysis, lipid peroxidation, Hb oxidation, and oxidation of -SH groups; these results are in line with those obtained in our study [69]. -SH groups are essential to preserve the integrity of the erythrocyte membrane; they protect cell structures from the action of free radicals by oxidizing and generating disulfide bridges [70]. The extract of MA leaves has also shown the ability to reduce the concentration of different radical species, such as DPPH, superoxide anion, hydroxyl radical, and to chelate and reduce iron. This could be linked mainly to the flavonoids present in the MA extract and also partially to the presence of DNJ. Studies in the literature showed that the alkaloid can neutralize the DPPH radical [71]. Among the flavonoids, quercetin, one of the main components of the extract, is taken up by erythrocytes and can affect the redox system of RBCs. It acts as an electron donor and interacts, at low concentrations, with the phosphatidylcholine and phosphatidylethanolamine heads, located in the cell bilayer, stabilizing it through hydrogen bonds [72,73].

Overall, the results obtained clearly demonstrate a peculiar (double) effect of the extract, which, on the one hand, confirming the data in the literature, reveals a protective action of the extract against the cell membrane. The extract could act from outside the membrane, perhaps accumulating on the lipid bilayer; in this context, phenolic compounds can exert antioxidant effects by neutralizing free radicals and inhibiting lipid peroxidation. On the other hand, the novelty of our research is the alteration of anion exchange and PTP-1B activity that could trigger destabilizing cell processes. These controversial effects of MA extract pave the way for new, deeper studies on the potential use of the intriguing properties of this plant.

## 5. Conclusions

In conclusion, our results confirm the antioxidant properties of Morus Alba leaf extract, probably due to its content of phenolic compounds and their derivatives. However, more extensive studies on RBCs, which fill some gaps in the literature, have shown controversial action.

Further studies are currently underway to fully understand the impact of the extract on the metabolic state of the erythrocyte.

## Figures and Tables

**Table 1:** Carbohydrates, phenolic compounds and flavonoid content in the extract of 100.0 μg/mL. Values were obtained using straight calibration lines made using standards (glucose for carbohydrates, gallic acid for phenolic compounds, and quercetin for flavonoid compounds, respectively).

Sample	Carbohydrates (mg Equivalents of Glucose)	Phenols (mg Equivalents of Gallic Acid)	Flavonoids (mg Equivalents of Quercetin)
100.0 μg/mL	0.970 ± 0.010	2.140 ± 0.037	2.200 ± 0.013

## Data Availability

The data that support the findings of this study are available from the corresponding author upon reasonable request.

## Supplementary Materials

The following supporting information can be downloaded at: https://www.mdpi.com/article/10.3390/biology14081005/s1, Figure S1: The influence of MA leaves extract on hemolysis values; Figure S2: Influence of MA leaves extract on met-Hb values; Table S1: Informations on volunteers subjects involved in the study.

## Abbreviations

The following abbreviations are used in this manuscript:

MA	Morus alba
RBCs	Red blood cells
NO	nitric oxide
ATP	adenosine triphosphate
PPP	pentose phosphate pathway
NADPH	reduced nicotinamide adenine dinucleotide phosphate
Hb	Hemoglobin
CDB3	cytoplasmic domain of band 3 protein
GE	glycolytic enzymes
FRAP	ferric reducing power
DPPH	2,2-diphenyl-1-picrylhydrazil
HEPES	2-[4-(2-hydroxyethyl) piperazin-1-yl]ethanesulfonic acid
H_2_O_2_	hydrogen peroxide
t-BHT	tert-butyl-hydroperoxide
AAPH	2,2-Azobis(2-methylpropionamidine) dihydrochloride
TBA	thiobarbituric acid
TBARS	thiobarbituric acid reactive substance
GSH	glutathione
