# Supplementary material for: Influence of Morus alba Leaves Extract on Human Erythrocytes
Source: Biology (Basel). 2025 Aug 5;14(8):1005. doi: 10.3390/biology14081005 (PMC12383867; doi:10.3390/biology14081005)
Supplement: Supplementary file 1 [file biology-14-01005-s001.zip › biology-3700226-supplementary.pdf]

Supplementary Materials:

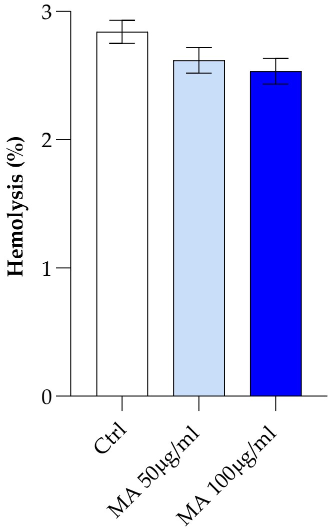

Figure S1. The influence of MA leaves extract on hemolysis values. As shown in the graph, MA leaves extract (50.0-100.0 µg/ml) did not cause a significant increase in the percentage of hemolysis. Mean ± SEM of at least N=3 independent experiments.

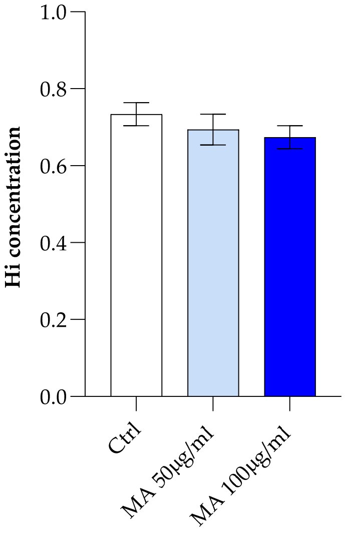

Figure S2. Influence of MA leaves extract on met-Hb values. As shown in the graph, MA leaves extract (50.0-100.0 µg/ml) did not cause significant changes in met-Hb concentrations. Mean ± SEM of at least N=3 independent experiments.

Table S1. Informations on volunteers subjects involved in the study.

Subjects characteristics	Range
number	20
age	27-30
sex	10 males and 10 femals
body mass index (BMI)	18.5 - 24.9 (normal weight)
health conditions	healthy subjects
drug assumption	no assumption
genetic factors	the subjects don't have pathological conditions due to genetic factors related to the study
